# Effectiveness of picture exchange communication system in developing requesting skills for children with multiple disabilities

**DOI:** 10.3389/fpsyg.2024.1434478

**Published:** 2024-12-20

**Authors:** Riyadh Khalid Alfuraih, Nabil Sharaf Almalki, Faisal Mohammed AlNemary

**Affiliations:** ^1^Department of Special Education, King Saud University, Riyadh, Saudi Arabia; ^2^Autism Center of Exellence, Riyadh, Saudi Arabia

**Keywords:** autism, intellectual disability, multiple disabilities, picture exchange communication system, request skills

## Abstract

This study evaluated the effectiveness of the Picture Exchange Communication System (PECS) in developing and generalizing requesting skills among children with multiple disabilities. The study was conducted at The Autism Center of Excellence, Riyadh, Saudi Arabia. This study included three participants age ranged from 4.5 to 6.5 years with intelligence quotient (IQ) scores ranging from 40 to 44. All participants were inrolled in Inclusive Education program. A training program based on PECS was implemented, and data collection involved recording participant responses and observations by external observers. The study followed an experimental approach, specifically utilizing Single-Subject Design (SSD) with a focus on Multi-Baseline Designs (MBD). The results of the study indicated that PECS facilitated the development of requesting skills in children with multiple disabilities, promoting skill retention and generalization across similar situations.

## Introduction

With more than one billion people, or approximately 1 in 7 individuals, living with some form of disability globally, and over 100 million children among them, the scale of this issue underscores the importance of addressing accessibility, inclusion, and support for individuals with disabilities across all sectors of society. These numbers highlight the significant impact disabilities have on individuals and communities worldwide, emphasizing the need for continued efforts to promote equality, empowerment, and opportunities for people of all abilities. It’s crucial to recognize that disabilities come in various forms, ranging from physical and sensory impairments to intellectual and developmental challenges, each requiring tailored support and accommodations to ensure full participation and inclusion in society (United Nations, 2022).

The prevalence of disabilities in the Kingdom of Saudi Arabia accounts for 7.1% of the total population. According to the General Authority for Statistics, there are approximately 1,445,723 individuals with disabilities out of a total of 32 million people. This comprises 52.2% of males and 47.8% of females. Among the disabled population, an estimated 102,865 individuals have multiple disabilities (General Authority for Statistics, Kingdom of Saudi Arabia, 2017; Saudi National Portal for Government Services).

Notably, individuals with intellectual disabilities and autism require particular attention. Research indicates that 40% of individuals with intellectual disabilities also experience developmental disorders. Furthermore, around 30% of individuals with autism spectrum disorder (ASD) are also diagnosed with intellectual disabilities. Additionally, many experience co-occurring developmental disorders, such as attention deficit hyperactivity disorder (ADHD) or communication challenges (Matson and Shoemaker, 2009). studies suggest that individuals with intellectual disabilities and autism often exhibit delays in verbal and linguistic behaviors (La Malfa et al., 2004).

Skinner’s theory asserts that obstacles related to language and communication take specific forms based on the prevailing conditions within the verbal community (Skinner, 1957). Consequently, these obstacles necessitate assistance from others. When a child makes a specific request, the reinforcement of that request becomes a motivating factor for the child. Requests hold great significance as they serve as the means through which speakers express their desires. The use of requests is strongly reinforced when the speaker’s request yields the desired results, often through unconditional reinforcement (Sundberg, 2017).

The study conducted by Mukhopadhyay and Nwaogu (2013) highlights the significant challenge involved in teaching non-verbal students with intellectual disabilities. It was observed that the utilization of augmentative and alternative communication systems was not widespread in Botswana. Several studies, including those by Chambers and Rehfeldt (2003), Curtis (2012), Van der Meer et al. (2012), and Achmadi (2015) have contributed valuable insights through literature review and theoretical frameworks.

Augmentative devices refer to specialized tools or equipment designed to enhance the communication abilities of individuals with speech or language impairments. These devices form a key component of Augmentative and Alternative Communication (AAC) systems, encompassing both low-tech solutions, such as communication boards and picture-based systems, and advanced high-tech devices, including speech-generating devices (SGDs). Their primary purpose is to complement or replace existing communication methods, thereby enabling users to effectively express their thoughts, needs, and emotions across diverse settings (Light and McNaughton, 2014).

Individuals with multiple disabilities and communication disorders necessitate alternative communication methods. Moreover, children with communication disorders can effectively employ the PECS (Skinner, 1957). Researchers in studies by Conklin and Mayer (2011), Strogonova and Piper (2018), and Al-Hamdi and Al-Farani (2019) have affirmed that individuals with multiple disabilities can be trained to use the PECS.

Chambers and Rehfeldt (2003) conducted a study to compare the effectiveness of manual sign language and the PECS in teaching request skills to adults with severe intellectual disabilities. Four adults were taught to request four desired items using each communication method. The results indicated consistent performance across all four areas using the picture exchange system for three out of the four participants. Among these three participants, two later demonstrated standard performance in requesting items using manual sign language.

In a study conducted by Curtis (2012), the objective was to determine the most effective approach for training children with autism spectrum disorder and intellectual disabilities in alternative communication methods. The study compared the effectiveness of manual sign language and the PECS in teaching six specific requests and increasing vocal behavior. The participants consisted of four children, aged (3) to (8), who had intellectual disabilities, autism spectrum disorder, or both, and exhibited limited communication skills. The results indicated that three participants successfully mastered requesting stimuli using the picture exchange system, while one participant achieved proficiency in requesting using manual sign language.

In Achmadi’s study (Achmadi, 2015), the aim was to identify the optimal method among three alternative communication approaches augmentative devices, manual sign language, and the picture exchange system for developing request skills. The study sample comprised four children, aged (4 to 5), with developmental disabilities, three of whom had autism and one had severe intellectual disabilities. The Vineland Adaptive Behavior Scale was utilized to assess the level of impairment, and a separate trial training strategy was employed. The training sessions were conducted in natural play environments at both home and school. The results indicated that three participants mastered the request skill using all three methods, while one participant achieved proficiency solely with the picture exchange system.

In contrast, Agius and Vance (2016) conducted a study to compare the effectiveness of two communication methods, namely the picture exchange system and the Sounding Board application on the iPad, in developing requesting skills in children under the age of six with autism spectrum disorder. The study involved a sample of three children. The results indicated that both the picture exchange system and the iPad with the Sounding Board application showed promise as effective tools for training requesting skills in preschool-aged children with autism.

Furthermore, Abu Delhom (2004) conducted a study to evaluate the effectiveness of the PECS in fostering communication skills in children diagnosed with autism spectrum disorder. The study included a sample of (20) children randomly divided into two groups: a control group of 10 children and an experimental group of (10) children. Post-measurement and a four-month follow-up revealed statistically significant differences in language communication skills between the experimental group, trained on the picture exchange system, and the control group. These results favored the experimental group, indicating the positive impact of the picture exchange system on communication skills.

Similarly, Ganz and Simpson (2004) conducted a study with the objective of examining the PECS and its effectiveness in expanding acquired vocabulary. The study sample consisted of three children diagnosed with autism spectrum disorder who shared similar characteristics. The participants were trained in various stages of the picture exchange system, including How We Communicate, Distance and Persistence, Discriminating Pictures, and Building Sentences. The results demonstrated a clear increase in vocabulary and a higher number of words used in a single sentence, supporting the effectiveness of the picture exchange system in achieving the objective of expanding vocabulary.

In a study conducted by Awaisat (2010), the objective was to determine the effectiveness of alternative and augmentative communication methods in enhancing receptive and expressive language skills among individuals with severe intellectual disabilities. The study compared two methods: the picture exchange system utilizing picture exchange communication and the gestural method using the Makaton program. The sample consisted of (20) participants. The results indicated significant improvements in both receptive and expressive language skills for the experimental group trained on the picture exchange system and the experimental group trained on the Makaton program. However, no statistically significant differences were observed between the picture exchange system and the Makaton gestural program when applied to the experimental group in terms of receptive and expressive language skills.

In another study, Park et al. (2011) investigated the impact of training parents in implementing the picture exchange system on the independent functional communication of three children diagnosed with autism spectrum disorder. The parents were taught the stages of the picture exchange system, including “How We Communicate,” “Distance and Persistence,” and “Discriminating Pictures.” The results demonstrated that the three children were able to independently exchange pictures, expanding their communication to various partners. Additionally, there was a limited improvement in speech production.

Similarly, Conklin and Mayer (2011) conducted a study to evaluate the effects of training in the picture exchange system using an MBD on three adults with developmental disabilities and severe communication impairments. Despite variations in training duration, all participants exhibited increased independent initiations after training on the picture exchange system. The results indicated a functional relationship between teaching the picture exchange system and enhancing independence, with continued improvements in independent initiations even after initial training. The participants trained on the picture exchange system also demonstrated increased initiation of requesting behaviors and subsequent improvements in their independence.

Boesch et al. (2013) conducted a single-case experimental study to compare the effectiveness of the PECS and the Speech Generating Device (SGD) in developing requesting skills for three children under the age of 12 with severe autism spectrum disorder. The study found that both communication interventions significantly increased the requesting behavior of the children, providing alternative means of communication. However, the children faced challenges in picture discrimination. Interestingly, there was no significant difference observed between the PECS and the SGD, indicating that both approaches are equally suitable for developing requesting skills.

In a similar vein, Strogonova and Piper (2018) investigated the effectiveness of the PECS in fostering communication and vocabulary development among children with intellectual disabilities. The study involved four children, fourteen parents, and eighteen teachers from the Riga Special Boarding School in Latvia. Through educational observation, the researchers assessed the skills and abilities of the participants at the beginning of the first semester and determined that the PECS was the most appropriate system for the sample. Importantly, all students readily accepted the introduction of the PECS. In addition, parents confirmed the utility of alternative communication tools, which could be actively incorporated into their children’s activities. Notably, the use of the PECS also led to a reduction in aggressive behaviors and improved the children’s ability to express their needs and emotions.

The current study is a valuable contribution to the scientific literature on the PECS within our Arab country. Its significance lies in its ability to assist researchers and individuals interested in addressing communication difficulties among children with multiple disabilities. Additionally, the study offers a theoretical contribution through the utilization of the MBD as an SSD. The study specifically focuses on children with multiple disabilities as participants.

During the researcher’s presence in daycare centers, they observed communication challenges among children with multiple disabilities. These challenges were attributed to the lack of specialized programs and services that prioritize the development of communication skills, particularly in the area of requesting. Motivated by these observations, the researcher decided to delve into this topic. The primary question addressed in this study is: What is the effectiveness of the PECS in developing requesting skills among children with multiple disabilities?

## Objectives

The study aims to achieve the following objectives:

1. Measure the effectiveness of the PECS in enabling children with multiple disabilities to acquire requesting skills.
2. Assess the children’s ability to maintain the acquired skill of requesting through the utilization of the PECS.
3. Evaluate the children’s capacity to generalize the skill of requesting across different contexts using the PECS.

## Method

In this study, a single subject design was chosen as the most appropriate method to address the research objectives and interpret the study variables accurately. The specific experimental methodology employed in this study is the MBD. In essence, the implementation of the intervention during the study is expected to bring about changes in the targeted behavior.

### Design

The MBD was utilized by the researcher to assess the effectiveness of the PECS in developing requesting skills among children with multiple disabilities. Among various design options, the Multiple Baseline Across Participants design was chosen. This design is particularly advantageous in its ability to accurately validate the presence of a functional relationship between the independent variable and the dependent variable (O'Neill et al., 2011).

### Sample

The study included a sample of three children with multiple disabilities who were enrolled in the Iiclusive Education Program at the Autism Excellence Center. The sample was purposefully selected and consisted of non-verbal children without hearing impairments, with intelligence quotient (IQ) scores ranging from 40 to 44 on the Stanford-Binet test. The participants’ chronological age ranged from 4.5 to 6.5 years. The specified criteria were carefully verified using a sample specification verification form, ensuring the accurate selection of the participating children (see Table 1).

**Table 1:** Data for the sample of children with multiple disabilities.

No	Child code	Chronological age	Mental age	IQ score	Medical diagnosis
1	B	6.3 years	2 years and 7 months	44 (Stanford-Binet Scale)	Intellectual disability and autism spectrum disorder
2	F	6.5 years	2 years and 6 months	40 (Stanford-Binet Scale)	
3	M	6.4 years	1 year and 8 months	41 (Stanford-Binet Scale)	

### Targeted behavior and training program using PECS

The current study provided a procedural description of the targeted behavior, which was identified as the requesting behavior, specifically requesting needs and reinforcements using the PECS. This behavior was selected based on reports from speech and language specialists and regular reports from the Autism Excellence Center, indicating expressive language difficulties and the inability of these children to make requests. The fourth phase of the PECS was utilized to develop the requesting skill.

- Overall program objective

The general objective of the program is to verify the effectiveness of using the PECS in developing the requesting skill for individuals with multiple disabilities.

B. Foundations of the training program

The training program was built upon several foundations, including:

1. Scientific foundation: the program was based on theoretical and scientific foundations related to the characteristics and features of individuals with multiple disabilities, as well as the utilization of the training protocol for the PECS.
2. Educational foundation: the educational foundation encompassed methods and strategies for the education and teaching of individuals with multiple disabilities.
3. Psychological foundation: psychological foundations of children with multiple disabilities were taken into consideration when developing the training program, including the following:

- Acknowledging individual differences among children with multiple disabilities.
- Addressing the needs of participating children with multiple disabilities.
- Considering the readiness and capacity of children with multiple disabilities to comprehend the training programs.
- Providing a safe physical and psychological environment for children with multiple disabilities.

4. Social foundation: social foundations were considered in building the training program, incorporating the following elements:

- Informing parents and specialists about the importance of developing the requesting skill for children with multiple disabilities.
- Collaborating with parents and teachers in implementing the training program.
- Obtaining consent from the parents of children participating in the training program.
- Focusing on communication skills between the researcher and children with multiple disabilities.

C. Program content

The program encompasses the following key components:

1. Clear explanation of the PECS and its significance in fostering the development of requesting skills among children with multiple disabilities.
2. Identification of the sequential steps involved in the implementation of the PECS to enhance the requesting skill of children with multiple disabilities.
3. Specification of the specific format through which children with multiple disabilities express their requests.
4. Definition of the researcher’s role in the intervention process, facilitating the children’s execution of the PECS steps.
5. Definition of the active role of children with multiple disabilities in successfully implementing the steps of the PECS.
6. Assessment of the children’s capacity to generalize their requesting abilities using the PECS with other individuals.
7. Determination of the number of training sessions, their timing, duration, and location within the program.

D. Program evaluation

Following the initial development of the training program, it underwent a comprehensive review by a panel of experts who provided valuable insights and feedback. Incorporating their suggestions, the training program was refined and enhanced to ensure its efficacy. The training program’s implementation mechanism, which utilizes the PECS to develop requesting skills, is explained, as depicted in the following Table 2:

**Table 2:** Mechanism for implementing the training program to develop requesting skills using the PECS.

Researcher	Child’s response	Researcher
Pre-questioning procedures: To facilitate the educational intervention, the researcher prepares the therapy room specifically for individual sessions with the child. The child is seated in front of a table, while the researcher occupies a position on the opposite side, ensuring optimal visibility and observation. The learning environment is carefully arranged to minimize distractions, including appropriate lighting and only essential materials displayed in front of the child. The picture exchange file is placed on a nearby table. The researcher initiates the interaction by asking the child: “What do you want?”	1) The child delivers a correct response by placing three pictures on the communication strip: a picture of “I,” a picture of “want,” and a picture representing the desired item or activity. The child then hands these pictures to the researcher.	1) Immediately, the researcher provides reinforcement to the child by lifting the communication strip in front of them and stating the phrase, “I want…” Additional verbal reinforcement may be provided, such as: “Excellent job, [child’s name]! You successfully requested the [name of the desired item/activity].” The researcher records the child’s response on the data sheet as a correct response, indicated by a “+” symbol on the data sheet.
	2) The child provides an incorrect response (prompted error).	2) The researcher provides full physical assistance (holds the child’s hand and guides it to the picture exchange file, places the “I + want + reinforcing picture” phrase, and pulls the communication strip, offering it to the child’s hand) as an incorrect response, indicated by a “-” symbol on the data sheet.
	3) The child provides a correct response with assistance (either by getting up from the seat only, getting up and heading towards the file only, getting up, heading towards the file, and placing some pictures, or getting up, placing the pictures, and not pulling the communication strip).	3) The researcher provides full physical assistance (holds the child’s hand and guides it to the picture exchange file, places the “I + want + reinforcing picture” phrase, and pulls the communication strip, offering it to the researcher) as a correct response with assistance, indicated by a “P” symbol on the data sheet.
	4) The child does not provide any response.	4) The researcher provides full physical assistance (holds the child’s hand and guides it to the picture exchange file, places the “I + want + reinforcing picture” phrase, and pulls the communication strip, offering it to the researcher) as no response, indicated by an “N” symbol on the data sheet.
	5) The child provides an incorrect response with assistance.	5) This response was not addressed during the study due to the researcher providing full physical assistance during training.

### Study tools

In order to achieve the objectives of the study, the following tools were developed and utilized:

1. Survey forms: were designed to verify the specifications of the sample, assess the target behavior, and gather information and data about the participating children.
2. Structured interview form: aimed to extract a comprehensive set of preferences reported by the specialist responsible for each child within the Autism Excellence Center. It is the approved form used within the center.
3. Independent observer’s observation form
4. Preference assessment test: was developed by reviewing relevant theoretical frameworks and previous studies that utilized preference assessment methods.
5. Reinforcer assessment test: was created by examining relevant theoretical frameworks and previous studies that focused on assessing reinforcers.
6. Data collection sheet: was developed based on a review of relevant theoretical frameworks and previous studies that employed discrete trial training.
7. Training pictures: were carefully selected based on the results of the preference assessment and reinforcer assessment.
8. Kit for the training program based on the PECS for children with multiple disabilities.
9. Interviews with the center’s management, specialists, and parents of the participating children.

The researcher presented the study tools to a panel of experts, who provided their opinions and recommendations. Necessary modifications were made to the tools based on their guidance, ensuring their validity and reliability for the study.

### Reliability and validity of study procedures

#### Reliability of study procedures

To ensure the reliability of the study procedures, an independent observer was appointed from the specialists within the Autism Excellence Center. This observer had the responsibility of conducting the observation process. Initially, the researcher verified the reliability of the entire session procedures by providing the independent observer with a meticulously prepared form. The form encompassed the specific application procedures implemented by the researcher during the training sessions with the participating children. These procedures comprised a total of (8) steps, which can be referred to in Appendix (10) for further details. The role of the independent observer was to mark “correct” if the step was executed and “incorrect” if it was not executed. However, an important condition was established: The observer’s markings should be in agreement with the researcher’s execution in (33%) of the total conducted sessions. The number of sessions corresponding to this percentage was determined using the following equation:

Total number of training sessions × 0.33.

Based on this, the researcher conducted a total of (7) training sessions, which is equal to (33%) of the sessions. The independent observer applied the form in these sessions. This calculation was done as follows:

$19^{*}0,33=6.27=7$

The researcher also ensured the reliability of each session’s procedures using the following equation:

${\left(\mathrm{number of executed steps}/\mathrm{total number of steps}\right)}^{*}\;100$

Thus, the percentage of reliability for implementing the PECS procedures for each participating child during a single session ranged between 87.5 and 100% over the course of seven training sessions for all children.

To calculate the overall average reliability of the study procedures, the following equation was used:

${\left(\begin{array}{l} \mathrm{sum}\;\mathrm{of session reliability percentages}\\ /\mathrm{number of observed sessions} \end{array}\right)}^{*}\;100$

Applying this equation, the overall average reliability for implementing the PECS procedures for all children across the observed sessions was calculated as follows:

687.5/7=98.21%

Therefore, the overall average value indicates a strong reliability in the researcher’s implementation of the PECS procedures, as depicted in the table for calculating the overall reliability percentage of the study procedures (see Table 3).

**Table 3:** The percentage of overall reliability for implementing the study procedures.

Data		Session number	Total number of steps	Sum of executed steps	Percentage of executed steps
Total number of training sessions	Number of sessions equivalent to 0.33 of the training sessions.	1	8	7	87.5
19	7	2		8	100
		3		8	100
		4		8	100
		5		8	100
		6		8	100
		7		8	100
Total sum					687.5
Mean					98.21%

#### Inter-rater reliability

The researcher assessed the inter-rater reliability between the observers (researcher and independent observer) to ensure the accuracy of recording children’s responses for each participating child during their training sessions using the PECS to develop requesting skills. The reliability of recording children’s responses was measured during (33%) of the sessions for each child using a “Child Response Recording Form” during the individualized program implementation. It was ensured that both the researcher and the independent observer recorded independently, without intervention or influence.

### Inter-rater agreement percentage for recording children’s responses

Recording Responses for the Implementation of the PECS: According to the researcher, the inter-rater agreement percentage for recording children’s responses was calculated using the following equation:

${\left(\begin{array}{l} \mathrm{number of agreements}/\mathrm{number of agreements}\\ +\mathrm{number of disagreements} \end{array}\right)}^{*}\;100$

Thus, the inter-rater agreement percentage for recording children’s responses to the implementation of the PECS procedures for each participating child ranged between 90 and 100% over the course of seven training sessions for all children.

To calculate the overall average of the inter-rater reliability for recording children’s responses in all observed sessions, the following equation was used:

$\begin{array}{l} \mathrm{sum}\;\mathrm{of inter}-\mathrm{rater agreement percentages}\\ /\mathrm{number of observed intervention sessions}=\mathrm{average} \end{array}$

Using this equation, the overall average inter-rater reliability for recording children’s responses to the implementation of the PECS procedures for all children and all observed sessions was calculated as follows:

680/7=97.14%

Therefore, the overall average value indicates a good indicator for the researcher’s recording of the independent variable’s results and data, influencing the dependent variable in this study. Refer to Table 4 for more information.

**Table 4:** The inter-rater agreement percentage during the recording of the study sample’s responses to the implementation of the PECS procedures.

Data		Session number	Number of agreements	Number of disagreements	Percentage
Total number of training sessions	Number of sessions equivalent to 0.33 of the training sessions.	1	9	1	90%
19	7	2	9	1	90%
		3	10	-	100%
		4	10	-	100%
		5	10	-	100%
		6	10	-	100%
		7	10	-	100%
Total sum					680
Mean					97.14%

#### Validity of study procedures

The researcher rigorously established the validity of the study procedures, ensuring that the utilization of the PECS was indeed responsible for the observed changes in children’s behavior related to requesting. This was accomplished through the following meticulous steps:

– To assess the effectiveness and consistency of the PECS, a well-designed MBD was implemented. This design encompassed a baseline phase followed by a targeted behavior intervention phase. Moreover, the system was systematically applied to all participants, focusing on the specific behavior of interest.
– Children with multiple disabilities, specifically those experiencing expressive language difficulties and an inability to make requests, were deliberately selected as the study’s sample from the Autism Excellence Center.
– The selection of the sample adhered to predetermined criteria aligned with the study’s objectives.
– A single independent variable, represented by the PECS, was deliberately introduced to all children with multiple disabilities. This was done to accurately evaluate its impact on enhancing the desired behavior of requesting.
– To maintain the integrity of the study, an agreement was established with the teachers and parents of the participating children, explicitly stating that no additional therapeutic interventions would be employed to influence the desired behavior.
– Precise measurement of the targeted behavior was attained through the implementation of a procedural definition.
– To enhance the reliability of the study, an independent observer was present alongside the researcher during designated study sessions. Their role was to diligently monitor and document the researcher’s implementation steps using a dedicated observation form.

### Statistical methods used

In this study, the researcher applied the MBD as an SSD, and utilized several statistical methods to analyze the data and derive meaningful results. These methods included reading frequency tables, calculating means, determining percentages of sample individuals, and conducting visual analysis of graphs (O'Neill et al., 2011). The purpose of employing these statistical techniques was to assess the effectiveness of the PECS in enhancing the requesting skill among children with multiple disabilities. Additionally, the performance of each student in the study sample was compared before and after the implementation of the study procedures.

## Study results and discussion

The first research question: What is the effectiveness of the PECS in developing requesting skills among children with multiple disabilities?

To address this research question, the researcher presented the results obtained from children with multiple disabilities as they demonstrated an increase in requesting behavior through the utilization of the PECS. The results can be summarized as follows:

### Bader

The child “Bader” demonstrated significant improvement in his requesting behavior, achieving the predetermined criterion of 100% on the graph over three consecutive sessions. This progress was observed during seven intervention sessions utilizing the PECS. Prior to the baseline phase, the researcher confirmed that “Bader” had not received prior training on the fourth stage of the PECS. To verify the desired behavior and assess its visibility to the specialist, the researcher provided a form to the responsible specialist at the Autism Excellence Center. This step was crucial in determining the need for appropriate intervention to enhance this behavior.

During the baseline phase, the researcher systematically measured the requesting behavior using a specific form, enabling a logical assessment of the presence of a functional relationship between the independent variable (Fahad’s System) and the observed changes in Bader’s requesting behavior.

### Fahad

The child “Fahad” demonstrated significant improvement in his requesting behavior, achieving the predetermined criterion of 100% on the graph over three consecutive sessions. This progress was observed during seven intervention sessions utilizing the PECS. Prior to the baseline phase, the researcher confirmed that “Fahad” had not received prior training on the fourth stage of the PECS. To verify the desired behavior and assess its visibility to the specialist, the researcher provided a form to the responsible specialist at the Autism Excellence Center. This step was crucial in determining the need for appropriate intervention to enhance this behavior.

At the beginning of the baseline phase, the researcher systematically measured the requesting behavior by making observations and utilizing a specific form tailored for this purpose. This procedure was implemented to logically evaluate the existence of a functional relationship between the independent variable (the PECS) and the dependent variable, which is the requesting skill. Its aim was to examine whether the implementation of the PECS had an observable impact on the child’s ability to make requests.

### Meshari

The child “Meshari” demonstrated significant improvement in his requesting behavior, achieving the predetermined criterion of (100%) on the graph over three consecutive sessions. This progress was observed during seven intervention sessions utilizing the PECS. Prior to the baseline phase, the researcher confirmed that “Meshari” had not received prior training on the fourth stage of the PECS. To verify the desired behavior and assess its visibility to the specialist, the researcher provided a form to the responsible specialist at the Autism Excellence Center. This step was crucial in determining the need for appropriate intervention to enhance this behavior.

During the baseline phase, the researcher carefully assessed the requesting behavior by making observations and utilizing a specific form designed for this purpose. This procedure was implemented to logically evaluate the existence of a functional relationship between the independent variable (the PECS) and the dependent variable, which represents the requesting skill.

**Figure 1:**
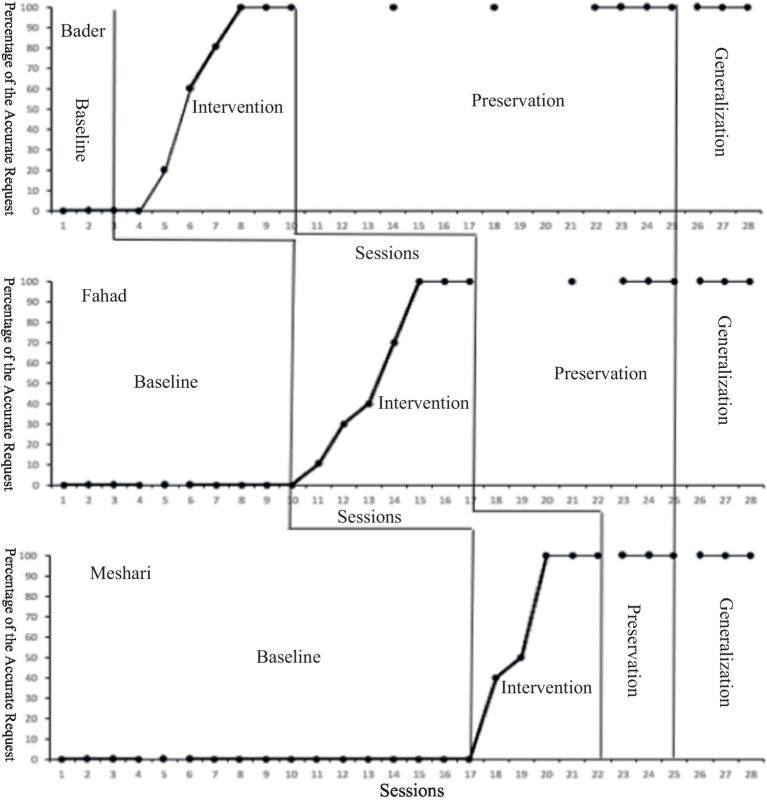
The percentage of requesting skills across 3 participants.

The previous results, summarized in Figure 1 and Table 5, clearly demonstrated a notable improvement in requesting behavior when children utilized the PECS. Remarkably, all children successfully met the predetermined criterion of achieving 100% on the requesting behavior graph for three consecutive sessions. The acquisition of this skill occurred through a series of intervention sessions, with each child requiring between five to seven sessions out of a total of 28 sessions, including maintenance and generalization sessions, as indicated in Table 6.

**Table 5:** Summary of data on requesting behavior frequencies among children with multiple disabilities.

S. no	Bader	Percentage	Fahad	Percentage	Meshari	Percentage
1	Baseline	0%	Baseline	0%	Baseline	0%
2	Baseline	0%	Baseline	0%	Baseline	0%
3	Baseline	0%	Baseline	0%	Baseline	0%
4	Intervention	0%	Baseline	0%	Baseline	0%
5	Intervention	20%	Baseline	0%	Baseline	0%
6	Intervention	60%	Baseline	0%	Baseline	0%
7	Intervention	80%	Baseline	0%	Baseline	0%
8	Intervention	100%	Baseline	0%	Baseline	0%
9	Intervention	100%	Baseline	0%	Baseline	0%
10	Intervention	100%	Baseline	0%	Baseline	0%
11	-	-	Intervention	10%	Baseline	0%
12	-	-	Intervention	30%	Baseline	0%
13	-	-	Intervention	40%	Baseline	0%
14	Preservation	100%	Intervention	70%	Baseline	0%
15	-	-	Intervention	100%	Baseline	0%
16	-	-	Intervention	100%	Baseline	0%
17	-	-	Intervention	100%	Baseline	0%
18	Preservation	100%	-	-	Intervention	40%
9	-	-	-	-	Intervention	50%
20	-	-	-	-	Intervention	100%
21	-	-	Preservation	100%	Intervention	100%
22	Preservation	100%	-	-	Intervention	100%
23	Preservation	100%	Preservation	100%	Preservation	100%
24	Preservation	100%	Preservation	100%	Preservation	100%
25	Preservation	100%	Preservation	100%	Preservation	100%
26	Generalization	100%	Generalization	100%	Generalization	100%
27	Generalization	100%	Generalization	100%	Generalization	100%
28	Generalization	100%	Generalization	100%	Generalization	100%

**Table 6:** Number of sessions required for children with multiple disabilities to acquire the requesting skill.

Child’s name	Number of sessions
Bader	7
Fahad	7
Meshari	5

## Discussion of results

The results obtained from all the children demonstrate positive outcomes and highlight their proficiency in utilizing the PECS to enhance their requesting skills. These results provide solid evidence for the effectiveness of the PECS in promoting the development of requesting abilities. Moreover, they underscore the robustness of the implemented training program, which was meticulously designed by the researcher to cater to the individual needs and preferences of the children. The program’s success can be attributed to the careful consideration of their preferred reinforcers and motivators.

While variations in performance and differences among the children were observed, these can be attributed to their individual abilities and capabilities. However, thanks to the careful selection and homogeneity of the sample, as well as the development of a structured training program tailored to the abilities of all the children, they were able to achieve the predefined criterion. This criterion entailed reaching a consistent 100% level of requesting behavior on the graph during three consecutive sessions.

Similarly, Van der Meer et al. (2012) conducted a study involving four participants under the age of 18 with intellectual disabilities or autism, who had limited verbal abilities and adequate hand motor skills for request training. The participants underwent a five-day training program, consisting of four daily sessions. The results demonstrated that all four participants achieved mastery in requesting their preferred items using both the picture exchange system and augmentative devices, whereas only two of them acquired the skill of requesting through manual sign language.

For example, both “Bader” and his peer “Fahad” replicated the requesting behavior using the PECS within the same time frame of seven days, spanning seven sessions. The similarity in the number of sessions can be attributed to the children’s concerted efforts to grasp the application mechanism effectively, coupled with their overall skill acquisition level. It is worth noting that during the intervention phase, Bader encountered a clear plateau in Session 4, where no progress was observed. As previously mentioned, this can be attributed to Badr having the least developed skill acquisition among the three children. However, after the fourth intervention session, Bader met the criterion and maintained this achievement throughout the subsequent maintenance phase, which commenced in Session 4. To clarify the methodology, after the last intervention session (Session 11), a waiting period of three sessions was implemented, and data for the maintenance session were collected in the fourth session. This approach was consistently followed for all maintenance sessions, including Sessions 23, 24, and 25. These sessions were conducted four days apart, and since no interventions occurred during these sessions, it becomes challenging to distinguish them in terms of behavior maintenance across all children.

Regarding the second child, “Fahad,” he successfully replicated the requesting behavior using the PECS within a consistent timeframe of seven days, spanning seven sessions, similar to his peer “Bader” as previously mentioned. Fahad achieved the predefined criterion during Session 4 of the intervention, and the same maintenance procedures were applied as with Bader.

Furthermore, the child “Meshari” demonstrated even faster progress, replicating the requesting behavior using the PECS within a shorter period of five days, encompassing five sessions. Meshari surpassed both Bader and Fahad in this regard. This exceptional performance can be attributed to Meshari’s enhanced ability to comprehend the application mechanism and overall skill acquisition. Notably, Meshari achieved the predefined criterion in the third session of the intervention, and the same maintenance procedures were followed as with Bader and Fahad.

The researcher suggests that the convergence among the children in terms of the number of sessions required during the intervention can be attributed to several factors, including intelligence level, mental age, and chronological age. Additionally, the researcher proposes that the convergence in the development of requesting skills using the PECS can be attributed to the remarkable ability of children with multiple disabilities to comprehend the system, despite their young age.

Three primary factors contribute to the children’s progress: joint assessment, reinforcement evaluation, and the number of training attempts before the intervention data collection session. All children were provided with a significant number of well-structured educational opportunities, facilitating the development of the targeted behavior. These factors directly contributed to the children with multiple disabilities’ ability to comprehend the training program’s defined steps and procedures.

The obtained results are in line with the results of Abu Delhom (2004), which emphasize the role of the PECS in developing communication skills. Similarly, they align with Awaisat’s study (Awaisat, 2010), which observed improvements in expressive language skills through the implementation of a picture exchange system. Consistent with Conklin and Mayer (2011), Boesch et al. (2013), and Strogonova and Piper (2018), this study demonstrates that participants who underwent training with the PECS experienced enhancements in their requesting abilities.

## Conclusion

The present study corroborates the results of (Abu Delhom, 2004; Ganz and Simpson, 2004; Awaisat, 2010; Conklin and Mayer, 2011; Grish, 2017; Strogonova and Piper, 2018; Al-Hamdi and Al-Farani, 2019; Al-Wabli and Al-Sajan, 2020; Almalki, 2021; and Alsayedhassan et al., 2021), supporting the notion that the utilization of the PECS leads to more effective communication skills. Furthermore, it aligns with Chambers and Rehfeldt (2003), Curtis (2012), and Van der Meer et al. (2012) emphasizing that children with multiple disabilities can successfully employ the PECS.

This study consisted of five main chapters. The first chapter covered the introduction, research problem, and the study questions. It also presented the study objectives, which aimed to investigate the effectiveness of using the PECS in developing and maintaining the requesting skills of children with multiple disabilities, as well as generalizing these skills. The study objectives also included measuring the effectiveness of training children with multiple disabilities to use the PECS for developing requesting skills. The chapter emphasized the theoretical and practical significance of the study, outlined its limitations, and defined relevant terms.

In the second chapter, the researcher discussed the literature review, focusing on the following dimensions: Multiple disabilities. Intellectual disabilities. Autism spectrum disorder. SSDs (MBDs). The chapter also reviewed previous studies, which were divided into two dimensions: Reviewing previous studies that addressed requesting skills. Reviewing previous studies that addressed the use of the PECS.

The third chapter outlined the methodology and procedures of the study. It included the study’s approach and its population, the variables and sample characteristics, the sample selection procedures, descriptions of the participants, the study’s tools and their application procedures, the validity and reliability of the study procedures and the statistical methods used in the study.

In the fourth chapter, the study results were presented, analyzed, and interpreted. This chapter included: analysis of the study’s instruments’ results and interpreting the results to answer the research questions.

The fifth chapter covered the study’s summary. It included the conclusion, the key results, the recommendations, suggestions for future research and limitations of the current study.

### Recommendations

Based on the results of the current study, the following precise recommendations are suggested:

– Provide training for teachers and specialists working with children who have multiple disabilities on the utilization of the PECS to enhance their requesting skills. This training should be designed to cater to the specific needs of professionals in this field.
– Foster collaboration among teachers, specialists, and families of children with multiple disabilities to generate solutions and suggestions that promote the development of desired behaviors. This collaborative effort can incorporate cognitive and behavioral systems and strategies, including the implementation of the PECS, which has demonstrated effectiveness in developing requesting skills as evidenced by this study. Integrating these strategies into inclusive education institutes and programs can effectively cultivate and reinforce desirable behaviors in children with multiple disabilities.
– Provide comprehensive training for children with multiple disabilities on various alternative communication systems in general, with specific emphasis on the PECS. This comprehensive training will significantly contribute to their learning and the development of their requesting skills, ultimately facilitating the achievement of educational goals for this particular group of children.

### Suggestions for further research

Several suggestions can be proposed to conduct future studies that build upon the results of the current study. These suggestions include:

– Conducting studies using the PECS to develop other skills, employing the methodology of SSDs.
– Conducting a study utilizing the PECS to develop the requesting skill in individuals with advanced stages of multiple disabilities, such as the fifth and sixth stages.
– Conducting a comparative study between the use of the PECS and another alternative communication system to determine which system is more effective in developing the requesting skill among children with disabilities.
– Conducting a study to assess the familiarity of teachers and specialists working with children with multiple disabilities regarding alternative communication systems.
– Conducting a study to identify the actual needs of teachers and specialists working with children with multiple disabilities regarding their acquisition of alternative communication systems.

### Study limitations

– Limited availability of recent Arabic studies on the topic of the current study, which necessitated the reliance on older studies in constructing the utilized training program for children with multiple disabilities.

– Limited sample size targeted in the current study due to the utilization of SSDs, which, according to theoretical literature, recommend a small sample size (O'Neill et al., 2011).

– Lack of training provided to children on the first three stages of the PECS using the same methodology employed in the current study.
– Participants in the current study had similar ages, making it challenging to find a homogenous sample in terms of characteristics and traits.

## Data Availability

The raw data supporting the conclusions of this article will be made available by the authors, without undue reservation.
